# A closed-loop negative feedback model for the pancreas: A new paradigm and pathway to a cure

**DOI:** 10.1097/MD.0000000000038802

**Published:** 2024-07-12

**Authors:** Thomas D. Dressel, Christine M. Custer

**Keywords:** closed-loop negative feedback model, control theory, pancreatitis

## Abstract

**Background and aims:**

To develop a model that describes how the pancreas functions, how the rate of synthesis of digestive enzymes is regulated, and finally what puts the pancreas to rest between meals.

**Methods:**

We applied the principals of control theory to previously published canine data to develop a model for how the canine pancreas functions. Using this model, we then describe the steps needed to apply this model to the human pancreas.

**Results:**

This new closed-loop negative feedback model describes what regulates digestive enzyme synthesis. This model is based on basolateral exocytosis of butyrylcholinesterase (BCHE) into the interstitial space. It is this level of BCHE * BCHE activity that controls the rate of canine pancreas digestive enzyme synthesis, and in the absence of stimulation from the vagus nerve, puts the pancreas to rest between meals.

**Conclusions:**

Finding secretagogue-specific inhibitory enzymes in the human pancreas that are analogous to BCHE in the canine, and blocking its associated receptors, may lead to a cure for human pancreatitis.

## 1. Introduction

Despite acute pancreatitis being described in the 17th century^[1]^ and chronic pancreatitis being first described in 1946,^[2]^ there is still no cure for this disease. Globally there were nearly 9 million cases of pancreatitis in 2015^[3]^ and over 132,000 deaths.^[4]^ The incidences increased by 24% and deaths increased by 21% from this disease between 2005 and 2015. More recent data for acute pancreatitis show the same troublesome trend. There was an increase from 1,727,789 cases in 1990 up to 2,814,972 cases in 2019.^[5]^ This is a major concern because the incidences continue to increase in high-income countries^[6,7]^ especially, and is most likely associated with the rise in rates of alcohol consumption and obesity.^[5]^ Pancreatitis remains one of the most common diseases in gastroenterology and treatment costs in the U.S. approached $8 billion in 2016.^[6]^ Additionally, acute pancreatitis and its reoccurrence leads to chronic pancreatitis, which can in turn lead ultimately to pancreatic cancer.^[7]^ While many of the proximate causes of acute pancreatitis, that are not caused by ductal obstruction,^[1]^ have been exhaustively described and are well known, the ultimate cause of pancreatitis other than from ductal obstruction is still a mystery. To this day, unfortunately, there is still no cure for pancreatitis, and treatment is similarly lacking except for palliative care,^[8]^ alcohol cessation counseling, and other treatments for symptoms.^[9]^ It is our and other’s assertions,^[7]^ that the lack of progress in finding a cure, or effective treatment, is because the regulation of the rate of digestive enzyme synthesis is not understood. No control mechanism has been proposed for the pancreas even though most biological systems have well understood feedback and control systems. Armed with the knowledge of what controls the rate of digestive enzyme synthesis, which is the subject of this paper, and what puts the pancreas to rest between meals, more effective treatments and possibly a cure for pancreatitis are within reach.

George E. Palade along with Albert Claude and Christian de Duve were awarded the Nobel Prize in 1974 for their work that laid the foundations of modern molecular cell biology. As part of that work specifically relating to the pancreas, Caro and Palade^[10]^ outlined the steps for digestive enzyme synthesis. That process begins with secretagogues occupying receptors on the acinar cells, reading of the genetic code for digestive enzyme synthesis, packaging of those enzymes by the Golgi apparatus, movement of the resulting zymogen granules to the apical region, and finally discharge into the pancreatic duct. That body of work greatly advanced our understanding of the structures, their functions, and processes within acinar cells. While that work was foundational, it did not address how those processes were regulated and what puts the acinar cells to rest between meals. Understanding the regulatory system is the next logical step in understanding how the pancreas functions.

**Figure 1.**
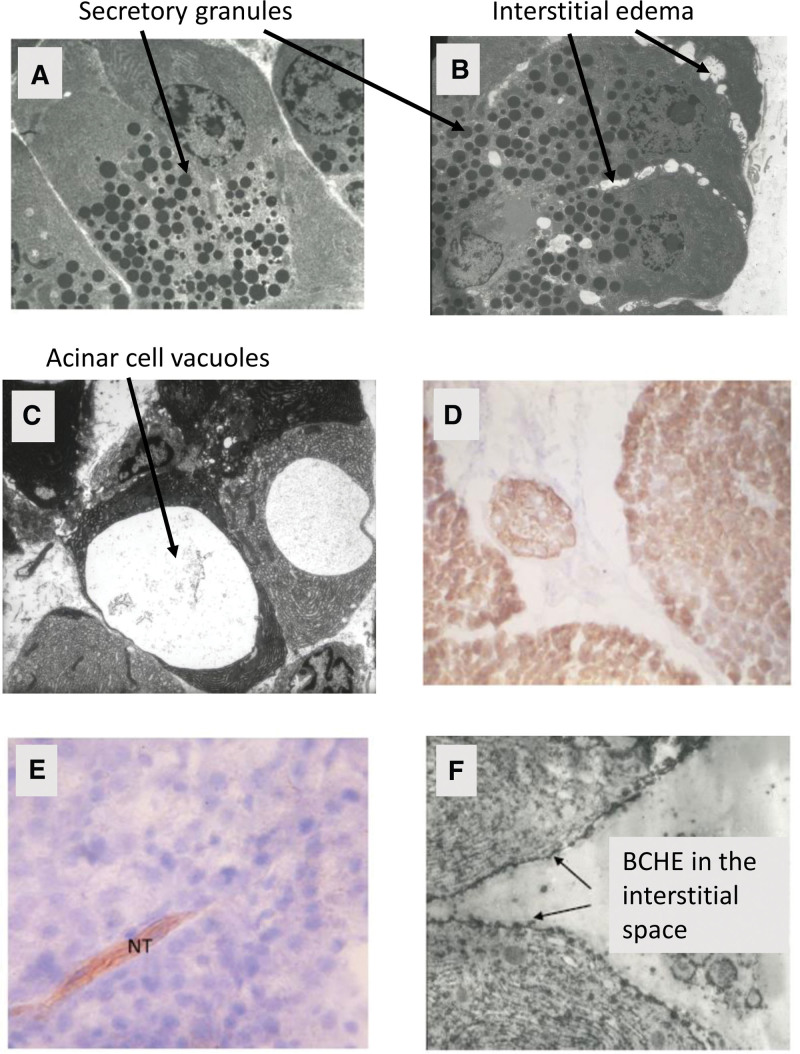
Electron microscope view of a normal pancreas (A), initial stages of interstitial edema at one hour after secretin + Diazinon-induced pancreatitis (B), large vacuoles in acinar cells at three hours after secretin + Diazinon (C), BCHE in canine acinar cells stained brown with Karnovsky stains (D), lack of BCHE except in the NT after atropine + secretin and Diazinon exposure (E), and a black and white electron micrograph of a resting state canine pancreas showing individual BCHE molecules not only within the acinar cells, but also in the interstitial space (arrows in [F], all data from Dressel et al^[11]^). BCHE = butyrylcholinesterase.

The pancreas is located in the abdomen, and its primary function is to secrete digestive enzymes when secretagogue receptors on the acinar cells are occupied, for example, by acetylcholine (ACh) from the vagus nerve as the sight and smell of food, and its consumption begins. These digestive enzymes include lipase, amylase, among others that break down fats, carbohydrates, and proteins. Once synthesized, these digestive enzymes are packaged by the Golgi apparatus into secretory vesicles for delivery to the duodenum via the pancreatic duct.^[10]^ It is only after these enzymes are delivered to the duodenum that they are activated. The packaging of these enzymes is important because it surrounds multiple copies of the digestive enzymes in a waterproof membrane that is prepared for apical exocytosis into the pancreatic duct. Each secretory vesicle package^[11]^ (Fig. 1A) behaves osmotically as a single particle and hence maintains osmotic stability within the pancreatic acinar cells. Hyperstimulation pancreatitis is characterized by vacuoles and edema along the basolateral cell walls^[11]^ (Fig. 1B and C) which likely results when the osmotic balance is upset because of an overproduction of digestive enzymes and the inability of the Golgi apparatus to properly package the digestive enzymes and deliver them to the duodenum.

In 1977 a 19-year-old patient was admitted to the University of Minnesota hospital.^[12]^ The patient was comatose and subsequently went into cardiac arrest but was resuscitated. After several causes for this illness were investigated, the patient was found to have pancreatitis; a pancreatic pseudocyst was discovered and was surgically drained into the stomach. After drainage of the pseudocyst, the patient’s amylase and lipase returned to normal within two days indicating that digestive enzyme synthesis was at normal levels. Diazinon (O,O-Diethyl O-[4-methyl-6-(propan-2-yl) pyrmidin-2-yl] phosphorothioate), a cholinesterase inhibiting insecticide, was identified as the toxic agent. This was the first time that an organophosphate cholinesterase inhibitor insecticide was documented and reported to have caused pancreatitis.^[12]^

To understand the mechanism for this poisoning, a series of experiments^[11]^ were conducted on dogs to elucidate the causes for Diazinon-induced pancreatitis. In brief, 8 dogs in each of 3 groups were intravenously injected with secretin at 2 units/kg h (control); secretin + Diazinon at 75 mg/kg; and secretin + Diazinon + atropine (200 µg/kg) with atropine injected immediately preceding the administration of Diazinon.^[11]^ The secretin + Diazinon-dosed dogs developed pancreatitis as evidenced by hyperlipasemia, hyperamylasemia, interstitial edema, and acinar cell vacuoles (Fig. 1B and 1C from^[11]^). The control-treatment group (secretin-only injection) did not develop pancreatitis and had normal levels of amylase and lipase in the serum. The dogs that received secretin + Diazinon + atropine also did not develop either hyperlipasemia or hyperamylasemia and vacuole formation was absent. The reason for this was that atropine blocked the ACh receptors and because of this, excessive amounts of digestive enzymes were not produced, and hence development of pancreatitis was prevented. Biopsies of canine acinar cells in the secretin + Diazinon + atropine dosage group were Karnovsky stained for butyrylcholinesterase (BCHE).^[13]^ Canine acinar cells normally show large amounts of BCHE activity (Fig. 1D from^[11]^), and in fact the level of BCHE, also known as pseudocholinesterase, in the canine pancreatic juice is at least 15 timed greater than present in canine serum.^[14]^ There was an absence of BCHE activity (Fig. 1E from^[11]^) in the acinar cells in the secretin + Diazinon + atropine dosage group because Diazinon destroyed the BCHE enzyme in the acinar cells. In the canine system, BCHE acts as a secretagogue-specific inhibitory enzyme (SSIE).

Closed-loop negative feedback control systems are the backbone of many biological systems. These closed-loop negative feedback control systems have two things in common: the ability to sample the output of the system being controlled and the ability to have a negative impact on the output of the system being controlled. Put another way, the control system constantly monitors and measures the process, compares the actual output with the desired output, and then adjusts the system to achieve the desired output. A more familiar and easier to understand closed-loop negative feedback control system is cruise control in an automobile. The cruise control sets the desired speed of the car, and the speedometer constantly monitors the speed. When the automobile encounters an upward incline, the speedometer senses a reduction in the speed, and the control mechanism automatically gives the engine more fuel to achieve and maintain the desired speed.

In this paper we expand on the ground-breaking work of Palade’s team and others, use the case study^[12]^ and associated experimental work^[11]^ described above, and take the next step which is to develop a model that describes what regulates the rate of digestive enzyme synthesis in the pancreas. The traditional paradigm for how the pancreas functions is an open-loop system, i.e., with no control mechanism to regulate the rate of digestive enzyme synthesis. This open-loop paradigm does not explain how the pancreas enters a resting state when there is no longer secretagogue stimulation and it also does not explain what causes over production of digestive enzymes. Over production of digestive enzymes is the root cause of pancreatitis when there is acinar cell damage caused by alcohol, insecticide poisoning, and other causes of pancreatitis.

In contrast, the new paradigm that we describe here is a negative feedback mechanism for controlling the rate of digestive enzyme synthesis, and specifically what puts the pancreas to rest between meals. We introduce the concept that BCHE which is synthesized in the canine pancreas along with digestive enzymes, is basolaterally transported into the interstitial space and is the basis for negative feedback regulation of the rate of synthesis of digestive enzymes; it also puts the canine pancreas to rest between meals. We know that basolateral exocytosis into the interstitial space of digestive enzymes occurs under hyperstimulation conditions or with excessive doses of a secretagogue.^[15,16]^ Basolateral exocytosis has also been documented to occur at physiological (normal) levels of stimulation as well,^[17]^ as evidenced by non-zero levels of lipase and amylase in the canine and human serum under physiologic level of stimulation.^[11]^ Basolateral exocytosis is shown histologically in the canine pancreas with the presence of BCHE clearly seen in the interstitial space (arrows, Fig. 1F). As a result, canine BCHE functions as an SSIE.

The objective of this paper is to present this new paradigm describing the regulatory processes in the canine pancreas, and by extension regulatory processes in the human pancreas. This new, closed-loop negative feedback model for the control of the rate of digestive enzyme synthesis is consistent with published data and explains not only the causes of acute and chronic pancreatitis, but it also specifically elucidates how damage to acinar cells, which can be caused by some diseases, excessive alcohol consumption, and poisoning by cholinesterase inhibitors, causes pancreatitis. This new paradigm provides the framework that can help guide subsequent research to find an effective treatment and a possible cure for pancreatitis, a common and devastating disease that has eluded all previous efforts to find a cure until now.

## 2. Material and methods

**Figure 2.**
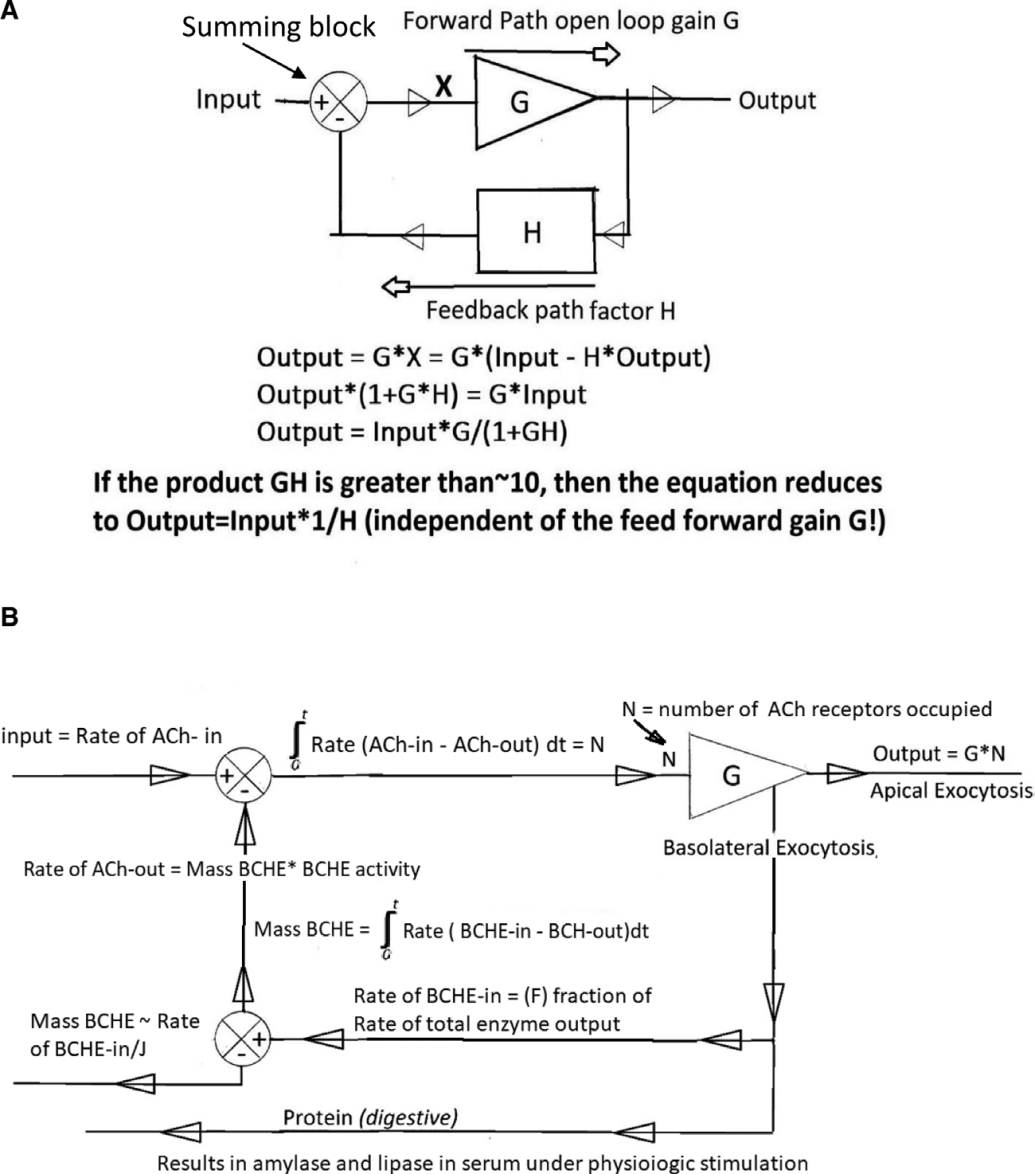
Block diagram of a basic control mechanism (A) and the mathematical model for the new closed-loop BCHE based negative feedback control model of the canine pancreas (B). BCHE = butyrylcholinesterase.

We used the principals of control theory and applied them to the existing data described above to develop this new paradigm that explains how acinar cells in the canine pancreas function and what the underlying regulatory mechanism is. We started with a mathematical model for a simple, generic closed-loop negative feedback control mechanism (Fig. 2A) and expanded it to include information from existing canine literature.^[11]^ In the simplified model, the output generated by G is continually monitored and modified by the feedback element (H), which has a negative influence on the input signal as shown with the negative sign in the summing block. The input signal as modified by the negative feedback element is represented by X. The key elements as they apply to the pancreas are as follows: G (the feed forward element) is the sensitivity of the acinar cells to ACh, N is the number of ACh receptors occupied, and H is the feedback element as stated above. We present both a mathematical and physiological description of this new paradigm to enhance understanding by the widest possible audience.

## 3. Results

### 3.1. Description of the mathematical elements in the canine negative feedback control model

In brief, the expanded model (Fig. 2B) starts with ACh coming into the interstitial space. The number of ACh receptors occupied (N) is determined by the time integral of the rate of ACh coming in (ACh-in) minus ACh going out (ACh-out, hydrolyzed). G is the sensitivity of the acinar cells to ACh and G * N results in the rate of total digestive protein synthesized of which a major portion is exported to the small intestine. A smaller proportion of the total digestive proteins synthesized, which includes BCHE, is basolateral transported into the interstitial space. F is the fraction of total enzyme output that is BCHE. The negative feedback element (H) is the mass of BCHE in the interstitial space which is the result of the rate of BCHE-in minus the rate BCHE lost (BCHE-out) by diffusion to the lymphatic system (J, Figure S1, Supplemental Digital Content, http://links.lww.com/MD/N116). The mass of BCHE times the BCHE activity then determines the rate of ACh-out and returns the system to the start of the cycle.

The rate of ACh-in sets the pace for the rate of digestive enzyme synthesis. The rate integrals are needed to account for differing rates of ACh-in, as well as differing rates of BCHE exocytosis into the interstitial space, and finally differing rates of BCHE lost to the lymphatics (Fig. 2B). The canine pancreas utilizes a variable rate of water soluble BCHE that is basolaterally transported into the interstitial space, at a rate that is a fraction (F) of the rate of total digestive enzyme synthesized and is secretagogue-dose dependent. This is the key signal that provides negative feedback control of the rate of synthesis of digestive enzymes (Fig. 2B). The mass of BCHE activity in the interstitial space is a balance between the rate of the mass of BCHE transported into the interstitial space and the rate of the mass of BCHE in the interstitial space that diffuses out to the lymphatic system. Calculation of the mass of BCHE in the interstitial space assumes 2 things that the rate of diffusion out is proportional to the mass of BCHE in the interstitial space (mass BCHE*J lost/min) and the rate of basolateral transport of the mass of BCHE into the interstitial space is the fraction (F) of the rate of total protein synthesis. The importance of linking this with total protein synthesis is that the mass of BCHE activity in the interstitial space is the negative feedback signal that allows the pancreas to constantly monitor the rate of synthesis of total digestive enzymes. It regulates the rate of total digestive protein syntheses by controlling the number of ACh receptors occupied (N, Fig. 2B), the more receptors that are occupied the faster digestive enzymes are synthesized, and conversely the fewer receptors that are occupied, the slower digestive enzymes are synthesized.

Simplifying the model by assuming steady state conditions, i.e., constant rate of ACh-in from the vagus nerve, removes the rate integrals. The integrals take into consideration the time lag that occurs from the time the ACh receptors are occupied, to when digestive enzymes and BCHE are synthesized and packaged for apical exocytosis into the pancreatic duct. The integrals also account for the time it takes for basolateral exocytosis of BCHE into the interstitial space and variability of the rate of ACh coming into the interstitial space during a meal. The steady state analysis requires that there is no variation in the rate of ACh into the interstitial space, and therefore, simplifies the analysis and makes it easier to understand how the pancreas really works. The following equations calculate the rate of digestive protein synthesis (Output). The closed-loop control mechanism transfer function is:

(1) Output/Input = G/(1 + GH)

(2) Output/ (Rate of ACh-in) = G/(1 + G * H) where H = (F/J) * (BCHE activity)

(3) Output = (Rate of ACh-in) * G)/[(G × F/J) * (BCHE activity)]

(4) Output = (Rate of ACh-in)/[(F/J) * (BCHE activity)]

(5) Output = G *N = (Rate of ACh-in)/[(F/J) * (BCHE activity)]

When (F/J) * (BCHE activity) is much greater than 10, the “1” in the denominator of the second equation can be ignored which results in the third equation above. In the case of the canine pancreas, G * H is almost always much greater than 10 because the activity of canine BCHE is such that a single molecule of BCHE can hydrolyze at least 5000 molecules of ACh/s. When G * H is much greater than 10, then the formula reduces to Output = Input * (1/H) and G becomes irrelevant. Note also that because G is present in the numerator and in the denominator of the third equation, the G’s cancel each other out which results in the fourth equation above. In the fifth equation, because Output = G * N where G = the sensitivity of the acinar cells to ACh and N = the number of ACh receptors occupied, the closed loop transfer function of the pancreas is independent of G, which is typical of most control mechanisms. As noted below, this closed-loop negative-feedback mechanism automatically compensates for changes in G. The equation results in a linear response to any changes in the steady state of the rate of release of ACh by the vagus nerve. For example, if the steady state of release of ACh into the interstitial space is doubled, then, while it will take some time to reach equilibrium again for the parameters listed above, eventually the rate of synthesis will double.

### 3.2. Physiological description of the closed-loop negative feedback model

**Figure 3.**
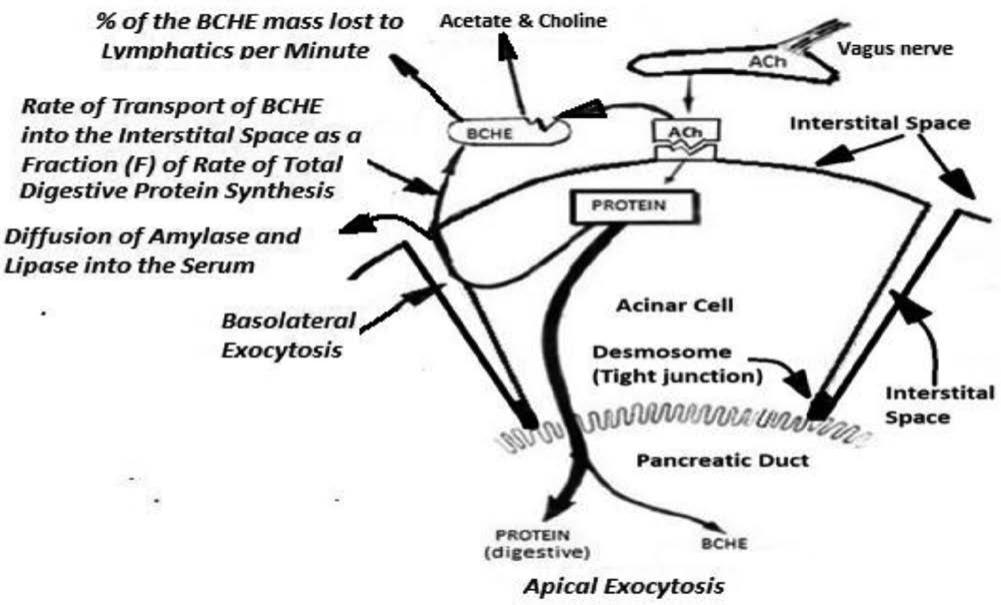
Diagram of the new closed-loop SSIE-based negative-feedback model with BCHE as the SSIE. Processes are italicized. BCHE = butyrylcholinesterase, SSIE = secretagogue-specific inhibitory enzyme.

To describe this new paradigm in non-mathematical terms, starting from a resting state the follow sequence is initiated: the vagus nerve releases ACh into the interstitial space and ACh begins to occupy the receptors (Fig. 3). The synthesis of digestive enzymes commences with most of the digestive enzymes being packaged by the Golgi apparatus and delivered to the duodenum. There is also basolateral exocytosis of small fraction of total digestive enzymes, as well as exocytosis of BCHE into the interstitial spaces. When there is an increase in the number of receptors occupied, the rate of total digestive protein synthesis increases resulting in an increase in the rate of BCHE transported into the interstitial space until mass of the BCHE activity which determines the rate of ACh hydrolyzed (broken down to acetate and choline) is equal to the rate of ACh released by the vagus nerve. This results in a stable number of ACh receptors occupied. When input from the vagus nerve deceases and then ceases between meals, the mass of BCHE activity in the interstitial space exceeds the rate of ACh-in from the vagus nerve, the number of receptors occupied decreases, thereby causing the rate of protein synthesis to decrease. This results in a lower rate of transport of BCHE into the interstitial space and less mass of BCHE activity in the interstitial space. With no more ACh coming in from the vagus nerve, the residual mass of BCHE activity in the interstitial space continues to remove the ACh from the receptors and the pancreas returns to a resting state with no receptors occupied and no digestive enzymes being synthesized. The rate of release of ACh into the interstitial space by the vagus nerve sets the rate of total digestive enzyme synthesis. Basolateral transport of the mass of BCHE into the interstitial space (mass of BCHE * BCHE activity) is the signal that allows the pancreas’ acinar cells to constantly monitor the rate of digestive enzyme synthesis and change that rate to match existing conditions; it is equivalent to cruise control in an automobile as explained above. In the canine model, BCHE is the SSIE. The pivotal role of the exocytosis of an SSIE, and its regulatory function, has gone unrecognized until now with the development of this model.

### 3.3. Explanation of how disruption to the negative feedback mechanism causes pancreatitis

Pancreatitis occurs when there is disruption, i.e. over production, during the process of digestive enzyme synthesis. When there is damage to the acinar cells, by alcohol for example, the sensitivity (G) of the acinar cells to ACh decreases. This will result in a lower rate of synthesis of digestive enzymes, a slower rate of transport of BCHE activity into the interstitial space, less mass of BCHE activity in the interstitial space, and therefore more ACh receptors being occupied. With more receptors occupied, the rate of synthesis of digestive enzymes increases. Up to a point, the pancreas control mechanism can automatically compensate for the damage to the acinar cells by giving the acinar cells the equivalent of more fuel in the automobile, i.e., an increased number of ACh receptors occupied. If the damage to the acinar cells is mild, the pancreas will continue to function as usual, but with an increased number of ACh receptors occupied. The Golgi apparatus can continue to package and deliver the digestive enzymes to the duodenum. If the damage is severe, however, the greater number of receptors occupied will cause overproduction of digestive enzymes, the Golgi apparatus ceases to package the enzymes appropriately, with the subsequent disruption of the osmotic balance that will cause hyperstimulation pancreatitis. In the case of a cholinesterase-inhibiting insecticide poisoning, when BCHE activity is inhibited or eliminated by exposure to a cholinesterase inhibitor, BCHE is not able to hydrolyze ACh and there is nothing to prevent all the ACh receptors from being occupied. This over production of digestive enzymes will always cause hyperstimulation pancreatitis. Mathematically, the denominator in the pancreas negative feedback transfer function, Output = G * N = (Rate of ACh-in)/[(F/J) * (BCHE activity)], goes to zero. Even though the denominator in the mathematical equation is zero, the number of receptors occupied cannot go to infinity because there are only a finite number of receptors on the acinar cells.

## 4. Discussion

### 4.1. Weight of evidence in support of the model

Because there are no empirical data to populate and validate the mathematical model, we used a weight of evidence approach to validate this model. Basolateral exocytosis of digestive enzymes and BCHE is essential to this model and had previously been reported to occur during hyperstimulation of the pancreas,^[15]^ or with excessive dosages of a secretagogue.^[16]^ In fact, basolateral exocytosis always occurs as a small fraction of the rate of total digestive enzyme synthesis, not just during hyperstimulation events or when acinar cells are damaged.^[17]^ Basolateral exocytosis is shown histologically at normal (physiological) levels of pancreatic function in the canine as evidence by residual BCHE being clearly seen in the interstitial space of a resting canine pancreas (arrows, Fig. 1F, Data from^[11]^). Low-level transport mechanism of digestive enzymes and BCHE in the interstitial space also explains why there are non-zero levels of amylase and lipase in the human and the canine serum in the absence of pancreatitis.^[11]^ This was previously unrecognized because of the difficulty of measuring these small concentrations under physiological levels of stimulation.

Another important discovery supporting this closed-loop negative-feedback model is documentation of the presence of high concentrations of BCHE^[11]^ in the canine pancreatic acinar cells and in pancreatic juice. As noted above, it is more than 15 times the concentrations that is found in canine serum.^[14]^ A critical question that was answered with research by Dressel et al^[11]^ was defining the function of BCHE in the interstitial spaces of the canine pancreas. Canine BCHE is a large molecule (350,000 Daltons) and metabolically expensive to synthesize, so it must serve a critical function to be produced in such large quantities. BCHE serves no digestive purpose because ACh is not part of the canine diet. BCHE actually serves two critical functions: the first is to regulate the rate of ACh-induced synthesis of digestive enzymes and the second is to put the pancreas to rest when there is no longer vagus nerve stimulation as described above. When BCHE activity is inhibited or destroyed, such as by administration of Diazinon^[11]^ in the canine, that regulatory mechanism becomes nonfunctional, and the canine always develops pancreatitis.

A fourth important discovery that validates this model is that BCHE in the canine, and by extension to an analogous SSIE in the human pancreas, is synthesized, stored, and secreted in an active form. In the canine pancreas BCHE, unlike the digestive enzymes which are activated only after they reach the duodenum, BCHE is active, even though it has never come into contact with the duodenum. It must have a function unrelated to the digestion of food. In the canine, the mass of BCHE activity in the interstitial space is the negative feedback mechanism that controls the rate of digestive enzyme synthesis.

A fifth critical element supporting this model was documenting the effect and significance of blocking the canine pancreatic cholinergic receptors with atropine. This prevented Diazinon-induced pancreatitis^[11]^ in the dog and may perform a similar function in humans. Blocking cholinergic or other receptors in the human pancreas temporarily with appropriate blocking agents will then stop synthesis of digestive enzymes and pancreatitis can either be prevented from developing further or be cured. This will represent a major break-through.

Development of this model was facilitated by the availability of standard techniques for staining BCHE and acetylcholinesterase (ACHE), in this case with Karnovsky staining methods.^[13]^ This allowed identifying those enzymes histologically at both the light and electron microscopic levels of magnification (Fig. 1D and E), and identification of BCHE in the interstitial spaces (Fig. 1F)

The canine pancreas, and by extension the human pancreas, showed itself to be amenable to application of the basic principles of control theory as detailed above. An ideal control mechanism requires that all cells, in this case the acinar cells, respond to a secretagogue in a reliably reproducible way. This is achieved because BCHE activity, which is at the center of this control mechanism in the canine pancreas and controls the rate of synthesis of digestive enzymes at the acinar cell level, is synthesized in a reliably reproducible way by the genetic code for this enzyme.

Finally, this model can also explain the mechanics behind other causes of pancreatitis such as acinar cell damage and how inhibition or destruction of the SSIE can result in hyperstimulation pancreatitis. Damage to the acinar cells results in decreased sensitivity of the acinar cells to ACh. This means that G, the sensitivity of acinar cells to ACh, will decrease, which will result in less BCHE activity in the interstitial space. That in turn causes N, the number of receptors occupied, to increase. That may result in hyperstimulation pancreatitis depending on the severity of damage. If BCHE activity is completely inhibited then there is nothing to prevent all of the acinar cells ACh receptors from being occupied, and this will always result in hyperstimulation pancreatitis.

### 4.2. Model applicability to the human pancreatitis

There is published evidence from studies of the human pancreas, although scant, that the model present here for the canine pancreas can also apply to the human pancreas with some modifications. There are three secretagogues in the human pancreas;^[18]^ two secretagogues in the human pancreas are associated with digestive enzyme synthesis. In one human study, Shiratori et al^[19]^ dosed human test subjects, who had acute pancreatitis, with increasing doses of loxiglumide, which is a cholecystokinin (CCK)-receptor antagonist. This treatment blocked CCK receptors on the acinar cells and thereby reduced excessive digestive enzyme synthesis. In that study however, the improvement in pancreatitis symptoms was only partial, a 36% improvement above that in the placebo group. That level of control is not insignificant, however, and it supports this closed-loop negative feedback model that we have developed and presented here. There are two possible reasons why this treatment was only partially effective. One reason is that the ACh receptor, the second human acinar cell receptor,^[20]^ was not blocked and some over synthesis of digestive enzymes continued. A second reason that the treatment might not have been more effective could be because loxiglumide is only a weak CCK-receptor antagonist and another CCK-receptor antagonist, such as L-364,718, which is a more potent CCK-inhibitor in the dog^[21]^ might have been more effective.

In a second study, Gabryelewicz et al^[22]^ investigated the effects of loxiglumide and atropine on duodenal volume and bicarbonate output in healthy individuals. Neither one of those endpoints was affected, however, similar to Shiratori et al,^[19]^ there was inhibition of pancreatic enzyme secretion in the loxiglumide-dosed individuals. Additionally, when atropine was injected, basal enzyme outputs were nearly completely inhibited. This result was almost identical to the canine dosage experiments by Dressel et al^[11]^ indicating that both the canine and human pancreas react similarly to atropine, i.e., a cessation or reduction of digestive enzyme synthesis.

The significance of these studies on humans was not recognized as a possible way to treat or cure pancreatitis because they either did not perform as expected^[19]^ or the experiment was designed to answer other questions.^[22]^ Both studies, however, support the applicability and validity of this model to humans that we developed and presented here for the canine pancreas. The significance and importance of these studies on humans^[20,22]^ might have been recognized and built upon with further studies had this larger context of the closed-loop negative-feedback model been available at that time.

## 5. Conclusion

The possible pathway to curing human pancreatitis, and testing this model, is predicated on the understanding that pancreatitis cannot occur if the pancreas and the syntheses of digestive enzymes are temporarily put to rest. This can be done by blocking the appropriate receptors, which will then temporarily prevent the synthesis of digestive enzymes. Because there is no BCHE synthesized in the human pancreas, there must be at least one SSIE in the human pancreas that is analogous to BCHE in the dog. It will be a serine protease (vulnerable to inhibition by a serine protease inhibitor) and it will be synthesized, stored, and secreted in an active form. Likely candidates for the SSIEs in the human pancreas are enzymes that eliminate the ability of ACh and CCK to function as secretagogues.^[18]^ The identification of SSIEs in human pancreatic juice would be one way to validate this model of a closed-loop negative feedback mechanism that we presented here for the canine pancreas. The identification of the target secretagogue of these human SSIEs will also establish the pathway to possibly curing pancreatitis when the agent(s) are found that can block the appropriate receptors, both the ACh- and CCK-receptors, just as atropine blocked the ACh receptor in the dog. At long last, a cure for pancreatitis is within reach.

## Author contributions

**Conceptualization:** Thomas D. Dressel.

**Formal analysis:** Thomas D. Dressel.

**Investigation:** Thomas D. Dressel.

**Project administration:** Thomas D. Dressel.

**Visualization:** Thomas D. Dressel, Christine M. Custer.

**Writing – original draft:** Thomas D. Dressel, Christine M. Custer.

**Writing – review & editing:** Thomas D. Dressel, Christine M. Custer.

## Supplementary Material


