# Supplementary figures and images for: A closed-loop negative feedback model for the pancreas: A new paradigm and pathway to a cure
Source: Medicine (Baltimore). 2024 Jul 12;103(28):e38802. doi: 10.1097/MD.0000000000038802 (PMC11245245; doi:10.1097/MD.0000000000038802)

## Slide 1
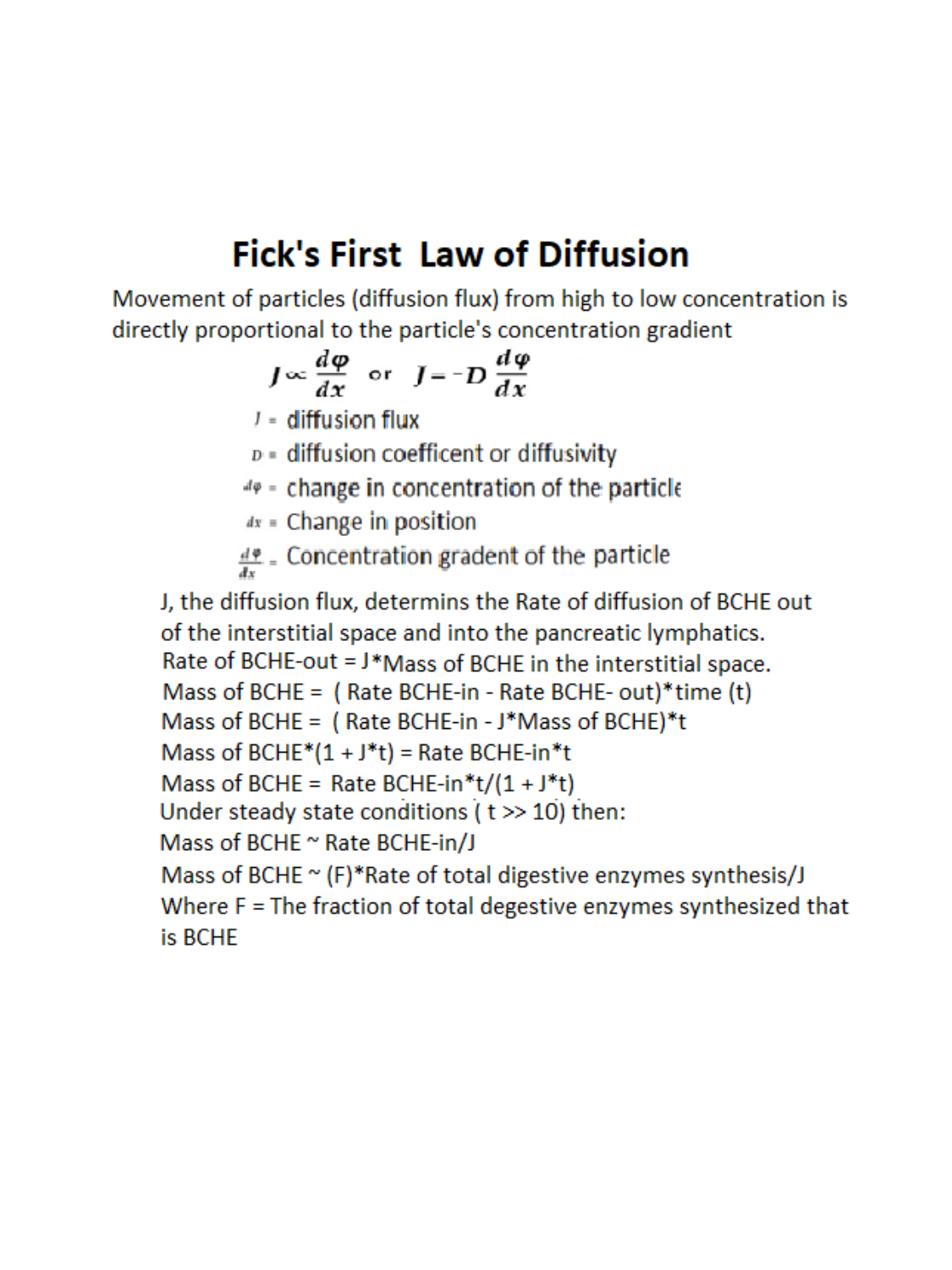

Supplement: Supplementary file 1 [file medi-103-e38802-s001.pptx]
